# Mental Illness Concordance Between Hospital Clinical Records and Mentions in Domestic Violence Police Narratives: Data Linkage Study

**DOI:** 10.2196/39373

**Published:** 2022-10-20

**Authors:** George Karystianis, Rina Carines Cabral, Armita Adily, Wilson Lukmanjaya, Peter Schofield, Iain Buchan, Goran Nenadic, Tony Butler

**Affiliations:** 1 School of Population Health University of New South Wales Sydney Australia; 2 School of Computer Science University of Technology Sydney Australia; 3 Hunter Medical Research Institute Newcastle Australia; 4 Institute of Population Health University of Liverpool Liverpool United Kingdom; 5 School of Computer Science University of Manchester Manchester United Kingdom

**Keywords:** data linkage, mental health, domestic violence, police records, hospital records, text mining

## Abstract

**Background:**

To better understand domestic violence, data sources from multiple sectors such as police, justice, health, and welfare are needed. Linking police data to data collections from other agencies could provide unique insights and promote an all-of-government response to domestic violence. The New South Wales Police Force attends domestic violence events and records information in the form of both structured data and a free-text narrative, with the latter shown to be a rich source of information on the mental health status of persons of interest (POIs) and victims, abuse types, and sustained injuries.

**Objective:**

This study aims to examine the concordance (ie, matching) between mental illness mentions extracted from the police’s event narratives and mental health diagnoses from hospital and emergency department records.

**Methods:**

We applied a rule-based text mining method on 416,441 domestic violence police event narratives between December 2005 and January 2016 to identify mental illness mentions for POIs and victims. Using different window periods (1, 3, 6, and 12 months) before and after a domestic violence event, we linked the extracted mental illness mentions of victims and POIs to clinical records from the Emergency Department Data Collection and the Admitted Patient Data Collection in New South Wales, Australia using a unique identifier for each individual in the same cohort.

**Results:**

Using a 2-year window period (ie, 12 months before and after the domestic violence event), less than 1% (3020/416,441, 0.73%) of events had a mental illness mention and also a corresponding hospital record. About 16% of domestic violence events for both POIs (382/2395, 15.95%) and victims (101/631, 16.01%) had an agreement between hospital records and police narrative mentions of mental illness. A total of 51,025/416,441 (12.25%) events for POIs and 14,802/416,441 (3.55%) events for victims had mental illness mentions in their narratives but no hospital record. Only 841 events for POIs and 919 events for victims had a documented hospital record within 48 hours of the domestic violence event.

**Conclusions:**

Our findings suggest that current surveillance systems used to report on domestic violence may be enhanced by accessing rich information (ie, mental illness) contained in police text narratives, made available for both POIs and victims through the application of text mining. Additional insights can be gained by linkage to other health and welfare data collections.

## Introduction

Domestic violence is defined as “any incident of threatening behavior, violence or (psychological, physical, sexual, financial, emotional) abuse between adults who are or have been an intimate partner or family member, regardless of gender or sexuality” [1], but it can also occur in other relationships including caregivers, guardians, parents, and a dependent person or those living together in a household such as flatmates [2]. Domestic violence is a global public health problem resulting in a significant economic and health burden on the community. In Australia, domestic violence is the leading cause of morbidity and mortality for women, surpassing risk factors such as obesity and smoking [3]. In 2018, 1 in 6 women and 1 in 16 men experienced physical or sexual violence by their current/former partner [4]. Research also shows that children exposed to domestic violence experience long-term effects on their development with an increased risk of poor mental health, learning difficulties, and behavioral problems [5].

Domestic violence has been linked to deaths, physical injuries ranging from minor traumas to those requiring hospitalization, depression, substance use, risky sexual behaviors, eating disorders, posttraumatic stress disorder, suicidal ideation and attempts, acts of self-harm, and exacerbation of psychotic symptoms [4,6-11]. Associations have been found between mental health conditions (eg, bipolar disorder, schizophrenia) and the perpetration of violence [12-18]. Evidence also suggests that people with mental illnesses are at a greater risk of victimization compared with those without [8,10,13,19-22]. Men and women with severe mental illness (eg, psychotic disorders) are 2-8 times more likely to experience any form of domestic abuse and have poor health outcomes (eg, suicide attempt, substance abuse) than the general population [19,23]. Thus, knowledge of the mental health of those involved in domestic violence may enable better prevention and intervention measures to be developed and implemented.

Administrative data collections represent a significant public resource [24,25]. Data linkage, the process that brings together individual-level data from different data collections, can provide valuable research insights and community benefits, although accessing them can be challenging as identified by the Australian Productivity Commission, and thus their use is often discouraged [26-29]. Data linkage offers a powerful, relatively inexpensive, population-wide, and accurate source of information to explore risk and protective factors. Linked administrative data have potentially very large sample sizes, access to the entire population information served by an agency, data on hard-to-reach populations, minimal loss to follow-up for certain outcomes, and a high level of external validity necessary for policy making [28,30]. These data are free from many of the measurement issues and biases that have been associated with qualitative research approaches (eg, in-depth victim interviews) that feature occasionally small sample sizes, are time consuming, and can have selection bias and high cost [31]. However, tapping into existing, routinely collected data using novel approaches such as text mining with subsequent linkage to different sectors’ data can complement these primary data collection methods in the area of domestic violence.

A common source of domestic violence data around the world are police records. In the Australian state of New South Wales (NSW), the NSW Police Force (NSWPF) attends and subsequently records details on thousands of domestic violence events each year. In NSW, a domestic violence event is defined as an incident of domestic dispute that involves any form of violence or abuse between a person of interest (POI)—an individual accused of perpetrating any form of violence or abuse toward another individual—and a victim. Information related to domestic violence events is recorded both as structured data (fixed fields, eg, covering demographic information such as name, date of birth, Aboriginal status) for the POI and the victim, and as a free-text narrative that describes details of the event (eg, cause, mental health status, threats of subsequent violence) based on the police officers’ observations and testimonies from the involved parties or the presence of witnesses (eg, neighbors, roommates, friends, family members).

Although the narratives may be used as an aide-mémoire for police officers and lawyers should the case proceed to court, to date they have not been used systematically for research and monitoring purposes due to their voluminous nature and the time taken to inspect and glean relevant information. We recently demonstrated that these event narratives contain rich information on perpetrators and victims of domestic violence such as mental illness [32], victim injuries, and abuse types [33]. We also demonstrated that such information could be extracted automatically, and that it could be used for population-wide domestic violence monitoring, surveillance, and prediction of domestic violence–related offences [34-36]. While other sources of domestic violence data might offer complementary information that will help to understand domestic violence, barriers to fully display its incidence and prevalence exist [37,38], leading to a potentially underrepresentation of this public health problem.

As mental illness is an important factor in domestic violence and we had previously identified a significant number of police events that contained mental illness mentions for both victims and POIs, the aim of this study is to examine the concordance between these mentions and corresponding diagnoses recorded by tertiary health services. This approach enables an assessment of the extent to which these 2 collections overlap and highlight their utility for domestic violence surveillance. This also allows an appraisal of relative merits, among which collection has more value to policy development in the domestic violence area.

## Methods

### Domestic Violence Police Events

We initially examined 492,393 police-recorded domestic violence events from January 2005 to December 2016 that were flagged in the fixed fields with 1 of the following tags: “domestic” as the offence type; “domestic violence related” as the associated factor of the police event; “spouse/partner (including ex-spouse/ex-partner),” “boy/girlfriend (including ex-boy/ex-girlfriend),” “parent/guardian (including step/foster),” “child (including step/foster),” “sibling,” “other member of family (including kin),” or “carer” as the relationship status between the victim and the POI. We note that domestic violence events can contain events that the police attended but no crime was committed.

### Text Mining Method and Normalization of Mental Illness Mentions

We previously developed a text mining methodology which we applied to domestic violence police event narratives that extracted mental illness mentions for POIs and victims [32]. The approach was implemented through the General Architecture for Text Engineering [39], which was chosen for its support of rule-based methods and easy manipulation of unstructured data. Rules were created after observing common lexical patterns that suggest a mental illness (Multimedia Appendix 1) for a POI (eg, “the POI is suffering from dementia”) or a victim (eg, “the victim was diagnosed with paranoid schizophrenia”), with semantic anchors relating the mention to either a POI (eg, “POI”, “person of interest”, “defendant”) or a victim (eg, “vic”, “PINOP—person in need of protection”, “victim”) in a sample of 200 police-recorded domestic violence events. This also included cases where:

- an unspecified mental illness was recorded (eg, “the defendant has mental health issues”);
- psychotropic drugs were used (eg, “the victim takes Valium”, “accused takes a number of antidepressants”), which might indicate a mental illness; these were categorized into 4 groups (antianxiety, antidepressants, neuroleptics, and antipsychotics); and
- individuals had traumatic brain injury or a drug prescription abuse (unspecified in the text regarding the medication), substance abuse (unspecified in the text regarding the substance), and drug-induced disorder (unspecified in the text regarding the drug).

The rules were combined with dictionaries that contained mental illness terms taken from the World Health Organization’s International Classification of Diseases, tenth revision (ICD-10) for Mental and Behavioral Disorders categories [40,41], including common abbreviations and synonyms. The methodology was fully evaluated against the manual annotations of mental illness mentions for POIs and victims by 2 experts (in domestic violence and neuropsychiatry, respectively, with an interannotator agreement of 90% [42]) in a random sample of 100 police-recorded domestic violence events, returning an average *F*_1_-score [43] of 84% (87.0% for POIs and 81.0% for victims); a detailed description of the methodology including its design, error analysis, and limitations has been published elsewhere [32].

As the extracted mentions ranged from specific (eg, “oppositional defiance disorder”) to general descriptions (eg, “behavioral problems”), these were mapped to ICD-10’s Mental and Behavioral Disorders categories using 3 levels (Multimedia Appendix 2) to conduct analysis of the results. Ambiguous case mapping was resolved by using the neuropsychiatry expertise of the fifth author (PS). A detailed analysis of the normalization process has been published elsewhere [32,34].

Although domestic violence events can have more than 1 POI or victim, the previous text mining methodology was unable to associate the extracted mental health “mention” with a specific POI or victim if more than 1 POI or victim was present in the same event. Thus, in this study we focused only on those events that included a single POI and a single victim. This resulted in 416,441 domestic violence events involving 214,148 unique POIs and 244,218 unique victims. By processing the associated narratives, 64,587 domestic violence events had at least one (unique) mention of mental illness for POIs and victims [32,34].

### NSW Health Records

Two sources of hospital records were used: the Admitted Patient Data Collection (APDC) and the Emergency Department Data Collection (EDDC). The APDC includes records for all hospital admissions from all NSW public and private hospitals and day procedure centers, whereas the EDDC provides information on presentations to emergency rooms in public hospitals in NSW. Each record in both collections includes a mental health diagnosis, the age of the patient at the time of diagnosis, and the start/end date of the admission. The EDDC contains diagnostic codes in the ICD-10 (eg, F10.0 stands for “mental and behavioral disorders due to use of alcohol, acute intoxication”), International Classification of Diseases, ninth revision (ICD-9; eg, 295.0 stands for “simple schizophrenia type”), and the Systematized Nomenclature of Medicine - Clinical Terms (SNOMED-CT; eg, 42344001 stands for “alcohol-induced psychosis”) formats, whereas the APDC has only ICD-10 codes. To ensure consistency with the data extracted from the police reports, the SNOMED-CT and ICD-9 diagnostic codes were mapped to the equivalent ICD-10 codes. For the conversion of the SNOMED-CT codes, we used a schema developed by the National eHealth Transition Authority, Australian Digital Health Service, where each SNOMED-CT code was assigned a corresponding ICD-10 code. The ICD-9 codes were converted through an online automatic converter, a process that was supervised by the fifth author (PS) [41]. Codes that could have several mappings to ICD-10 were assigned to 1 code only based on the expertise of the fifth author (PS).

The respective window of these 2 collections involved records from July 2001 to September 2018 (APDC) and from January 2005 to September 2018 (EDDC), which covered the period of the domestic violence event data set (January 2005 to December 2016).

### Linkage Between Police Records and NSW Health Records

**Figure 1:**
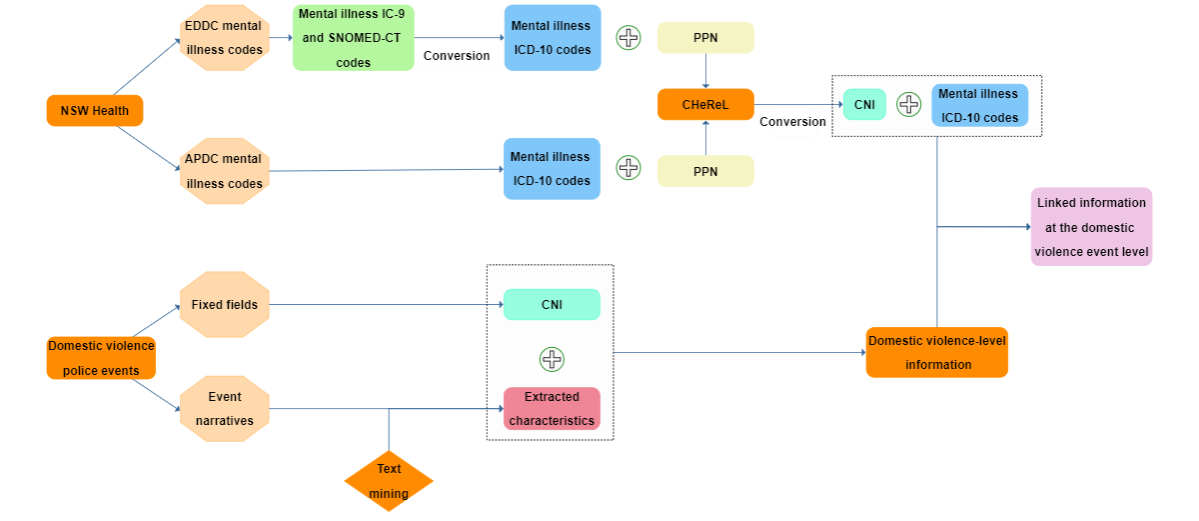
An overview of the data used in the linkage process. CNI is the police’s unique number for each individual involved in a domestic violence event. PPN is the converted CNI that is required to link the individuals of a police record with the EDDC and APDC data collections. APDC: Admitted Patient Data Collection; CHeReL: Centre for Health Record Linkage; CNI: Criminal Number Index; EDDC: Emergency Department Data Collection; ICD-9: International Classification of Disease, ninth Revision; ICD-10: International Classification of Disease, tenth Revision; NSW: New South Wales; PPN: Project Person Number; SNOMED-CT: Systematized Nomenclature of Medicine - Clinical Terms.

The police’s unique Criminal Number Index (CNI) for each individual involved in a domestic violence event was converted into a unique Project Person Number (PPN) by the Centre for Health Record Linkage to link these individuals with the EDDC and APDC data using probabilistic record linkage [44]. An overview of the data linkage process including the application of the text mining methodology on the domestic violence police narratives is shown in Figure 1. A detailed description of all the information (ie, text mined, fixed fields, and external mental health diagnoses) used to describe a domestic violence event is provided in Multimedia Appendix 3.

### Concordance Analysis

To examine concordance between the police and secondary care systems, we used descriptive statistics. It should be noted that an individual might have more than 1 diagnosis or police mental illness mentions. Thus, at an event level, concordance was defined as an exact agreement on a mental illness diagnosis code between a police mention and a hospital record for an individual (ie, how many events have the same code in police mentions and hospital records irrelevant of how many other diagnosis codes might be associated for this particular individual at this event). Concordance for a specific mental illness followed the same approach (ie, how many events had an agreement on a specific mental illness code between police mentions and hospital records). We used only the first level of ICD-10 to inspect the concordance. This means that more specific mentions from either side (eg, a depression mention from police narratives or a bipolar disorder diagnosis from a hospital admission matched at the first level of ICD-10, which was “mood [affective] disorders”) were aggregated to the first level for reporting purposes.

Given that the dates for hospital admissions and emergency department presentations were unlikely to align in a temporal sense with the domestic violence event, we used window periods of varying lengths (ie, 1, 3, 6, and 12 months) before and after the event to examine the concordance between the extracted (police mentions) and diagnostic information (hospital records). This approach was adopted to examine existing mental health contacts with secondary and tertiary health care systems. Those conditions seen by primary care providers or parts of the health care system other than hospitals would not have been captured by our data linkage.

### Ethics Approval

Permission to access the domestic violence events was granted by the NSWPF following ethics approval from the University of NSW Human Research Ethics Committee (approval number HC16558). Approval was also granted by the NSW Ministry of Health (approval number HC16558) to access information contained in the APDC and EDDC.

## Results

### Overview

Using no window period for the linkage between the hospital and police records for 416,441 domestic violence police events, the total number of events with a hospital record of mental illness was 142,324/416,441 (34.18%) and the number of events with mental illness police mentions was 64,587/416,441 (15.51%; Table 1).

**Figure 2:**
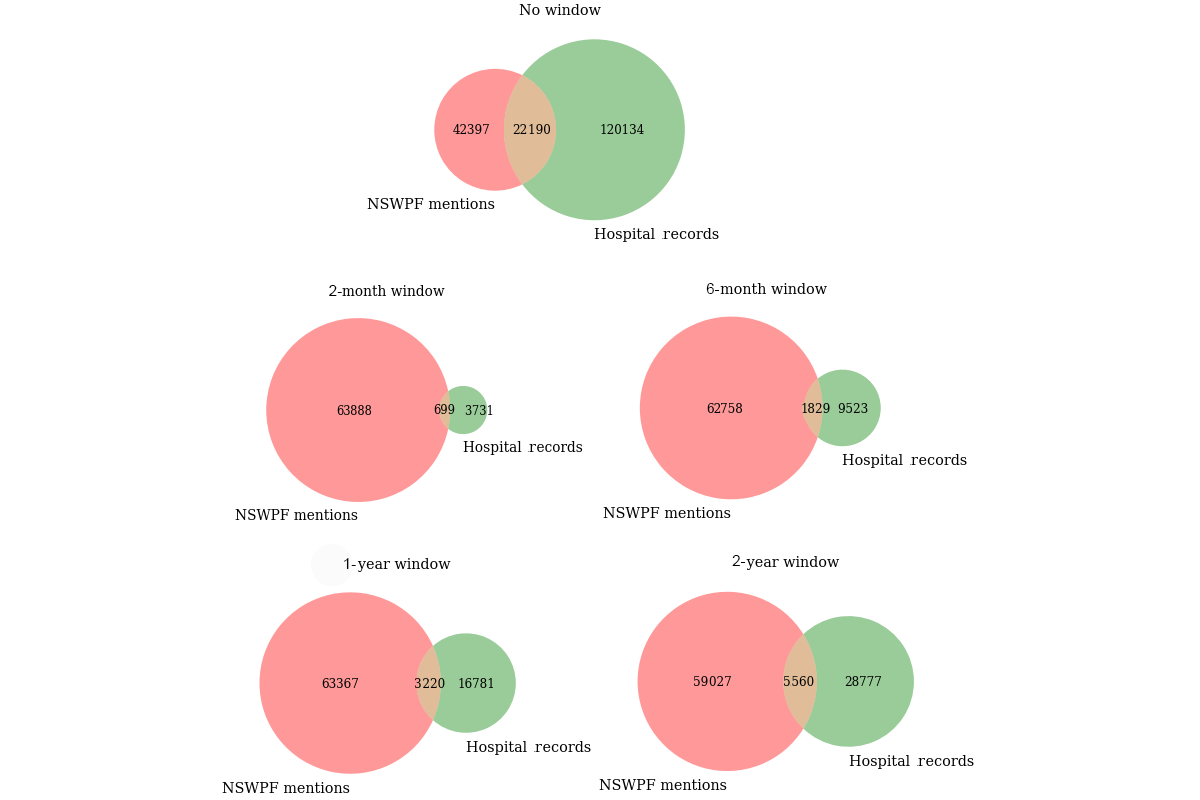
Number of domestic violence police events with a police mention and a hospital record of mental illness using various window periods (ie, 1, 3, 6, and 12 months before and after the domestic violence event), and no window period. NSWPF: New South Wales Police Force.

Using a 2-year window period (ie, 12 months before and after the domestic violence event), the number of events with a corresponding hospital record with any mental illness was reduced to 34,337/416,441 (8.25%; Table 2). Predictably, the larger the window period used, the more overlap there was between the police and hospital records related to mental illness. The least overlap was seen when applying the shortest (ie, 1 month before and after the domestic violence event) window period, where only 699 police and hospital systems recorded a mental illness for either the POI or victim (Figure 2).

**Figure 3:**
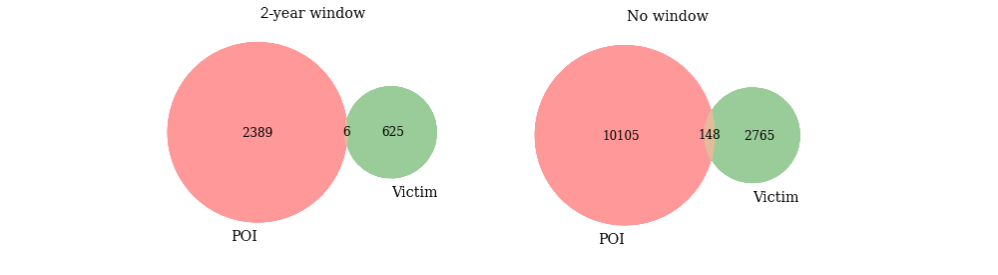
Number of domestic violence police events with a police mention and hospital record of mental illness for a POI and a victim using a 2-year window (ie, 12 months before and after the domestic violence event occurred) and no window period. POI: person of interest.

Using a 2-year window, a total of 5560 domestic violence events had both a hospital record and a police mention of mental illness for either the POI or victim (Table 2). Regardless of the diagnosis, in 3020/5560 (54.32%) events the mental illnesses recorded by the police and hospital systems agreed in terms of having a diagnosis present for the POI (2395/3020, 79.30%) and victim (631/3020, 20.89%; Figure 3). However, 2540/5560 (45.68%) events reported mental health information for different individuals (ie, the police recorded a mental illness mention in an event for a POI, whereas the linked hospital record of mental illness for the same event was attributed to the victim).

**Table 1:** Number of domestic violence events with or without a recorded mental illness in the APDC^a^/EDDC^b^ collections or a reported (unique) mention from the police narratives—no window period applied (N=416,441)^c^.

APDC/EDDC diagnosis code	Police mental illness mentions, n	
	Yes (n=64,587)	No (n=351,854)
Yes (n=142,324)	22,190	120,134
No (n=274,117)	42,397	231,720

^a^APDC: Admitted Patient Data Collection.

^b^EDDC: Emergency Department Data Collection.

^c^“Yes” refers to the number of domestic violence events/hospital records that have a mental illness mention/diagnosis.

**Table 2:** Number of domestic violence events with or without a recorded mental illness in the APDC^a^/EDDC^b^ collections or a reported mention from the police narratives; 2-year window period applied (ie, 12 months before and after an event occurred; N=416,441)^c^.

APDC/EDDC diagnosis code	Police mental illness mentions, n	
	Yes (n=64,587)	No (n=351,854)
Yes (n=34,337)	5560	28,777
No (n=382,104)	59,027	323,077

^a^APDC: Admitted Patient Data Collection.

^b^EDDC: Emergency Department Data Collection.

^c“^Yes” refers to domestic violence events/hospital records that have a mental illness mention/diagnosis.

### Concordance

Using a 2-year window period, the concordance between hospital and police records for POIs was in only 382 events (382/2395, 15.95%), whereas for victims it was in 101 (101/631, 16.01%) events. The concordance rate was highest for mental and behavioral disorders due to psychoactive substance use when taking into consideration the total number of police events with a respective mention (POIs: 151 events out of 5616, 2.69%; victims: 26 events out of 1098, 2.37%; Table 3).

When no window period was applied, the concordance rate between police and hospital records increased: for POIs it was 19.56% (2005/10,253), whereas for victims it was 18.50% (539/2913). Despite mood disorders having a higher number of events that saw concordance between police and hospital records, their concordance rate was lower in both POIs (756/11,985, 6.31%) and victims (233/4108, 5.67%), whereas mental and behavioral disorders due to psychoactive substance use had the highest concordance rate (692/5616, 12.32% for POIs and 115/1098, 10.47% for victims; Table 4).

**Table 3:** Number of domestic violence police events with the same mental illness diagnosis (presented as ICD-10^a^ mental illness groups) in the police and hospital systems for POI^b^ and victims—2-year window period applied.

Mental illness group (ICD-10)	Total^c^ (POI)	Number of events with a concordance^d^ (POI)	Concordance rate, %	Total^c^ (victim)	Number of events with a concordance^d^ (victim)	Concordance rate, %
Mental and behavioral disorders due to psychoactive substance use	5616	151	2.7	1098	26	2.4
Mood (affective) disorders	11,985	110	0.9	4108	35	0.9
Schizophrenia, schizotypal, delusional, and other nonmood psychotic disorders	4529	50	1.1	878	3	0.3
Anxiety, dissociative, stress related, somatoform, and other nonpsychotic mental disorders	2855	29	1.0	1832	21	1.1
Unspecified mental disorder	21,839	28	0.1	4198	10	0.2
Behavioral and emotional disorders with onset usually occurring in childhood and adolescence	7097	9	0.1	1721	2	0.1
Intentional self-harm	2694	6	0.2	821	2	0.2
Disorders of adult personality and behavior	1067	3	0.3	355	1	0.3
Pervasive and specific developmental disorders	1338	1	0.1	401	1	0.2
Intellectual disability	1196	0	0.0	782	1	0.1

^a^ICD-10: International Classification of Disease, tenth revision.

^b^POI: person of interest.

^c^Total number of events with a text-mined mention of mental illness

^d^Number of events with an agreement between a text mining (police) mental illness mention and a mental health (hospital) diagnosis code.

**Table 4:** Number of domestic violence police events with the same mental illness diagnosis (presented in ICD-10^a^ mental illness groups) in the police and hospital systems for POI^b^ and victims—no window period applied.

Mental illness group (ICD-10)	Total^c^ (POI)	Number of events with a concordance^d^ (POI)	Concordance rate, %	Total^c^ (victim)	Number of events with a concordance^d^ (victim)	Concordance rate, %
Mood (affective) disorders	11,985	756	6.31	4108	233	5.7
Mental and behavioral disorders due to psychoactive substance use	5616	692	12.3	1098	115	10.5
Unspecified mental disorder	21,839	187	0.9	4198	44	1.0
Anxiety, dissociative, stress related, somatoform, and other nonpsychotic mental disorders	2855	181	6.3	1832	101	5.5
Schizophrenia, schizotypal, delusional, and other nonmood psychotic disorders	4529	133	2.9	878	32	3.6
Behavioral and emotional disorders with onset usually occurring in childhood and adolescence	7097	85	1.2	1721	13	0.8
Intentional self-harm	2694	31	1.2	821	10	1.2
Disorders of adult personality and behavior	1067	21	2.0	355	4	1.1
Intellectual disability	1196	5	0.4	782	2	0.3
Pervasive and specific developmental disorders	1338	3	0.2	401	3	0.7
Mental disorders due to known physiological conditions	496	2	0.4	579	1	0.2
Symptoms and signs involving cognition, perception, emotional state, and behavior	160	1	0.6	72	1	1.4

^a^ICD-10: International Classification of Disease, tenth revision.

^b^POI: person of interest.

^c^Total number of events with a text-mined mention of mental illness.

^d^Number of events with an agreement between a text mining (police) mental illness mention and a mental health (hospital) diagnosis code.

### Events With a Police Mention and No Hospital Record of Mental Illness

In a 2-year window period, 59,027/416,441 (14.17%) domestic violence events had a police mention but no hospital record of mental illness (Table 2), with 51,025/59,027 (86.44%) event mentions related to POIs and 14,802/59,027 (25.08%) to victims. Unspecified mental disorders (generic mentions of mental illness in the narrative text such as mental disorder, some form of mental illness) were the most common in POI (20,890/51,025, 40.94%) and victims (4043/14,802, 27.31%), followed by mood disorders (11,427/51,025, 22.39%, in POIs and 3938/14,802, 26.60%, in victims). Anxiety disorders in victims (1752/14,802, 11.84%) had more than double the rate when compared with that of the POIs (2731/51,025, 5.35%; Table 5).

**Table 5:** Number of domestic violence police events with a mention of mental illness for POIs^a^ and victims without a respective hospital record—2-year window period applied (n=51,025)^b^.

Mental illness group (ICD-10^c^)	Events (POI; n=51,025), n (%)	Number of events (victim; n=14,802), n (%)
Unspecified mental disorder	20,890 (40.94)	4043 (27.31)
Mood (affective) disorders	11,427 (22.39)	3938 (26.60)
Behavioral and emotional disorders with onset usually occurring in childhood and adolescence	6791 (13.31)	1645 (11.11)
Mental and behavioral disorders due to psychoactive substance use	5360 (10.50)	1064 (7.19)
Schizophrenia, schizotypal, delusional, and other nonmood psychotic disorders	4306 (8.44)	840 (5.67)
Anxiety, dissociative, stress related, somatoform, and other nonpsychotic mental disorders	2731 (5.35)	1752 (11.84)
Intentional self-harm	2577 (5.05)	783 (5.29)
Substance abuse	2177 (4.27)	302 (2.04)
Pervasive and specific developmental disorders	1278 (2.50)	378 (2.55)
Intellectual disability	1143 (2.24)	737 (4.98)
Disorders of adult personality and behavior	1021 (2.0)	339 (2.29)
Injury of unspecified body region	658 (1.29)	208 (1.41)
Traumatic brain injury	531 (1.04)	197 (1.33)
Mental disorders due to known physiological conditions	479 (0.94)	557 (3.76)
Medications: antidepressants	311 (0.61)	105 (0.71)
Symptoms and signs involving cognition, perception, emotional state, and behavior	154 (0.30)	68 (0.46)
Medications (antipsychotics)	104 (0.20)	13 (0.09)
Medications for anxiety	73 (0.14)	20 (0.14)
Other degenerative diseases of the nervous system	51 (0.10)	43 (0.29)
Chromosomal abnormalities, not elsewhere classified	46 (0.09)	30 (0.20)
Unspecified drug-induced disorders	43 (0.08)	0 (0.0)
Behavioral syndromes associated with physiological disturbances and physical factors	22 (0.04)	17 (0.1)
Systematic atrophies primarily affecting the central nervous system	10 (0.02)	6 (0.04)
Diseases of the nervous system	3 (0.01)	2 (0.01)
Drug prescription abuse	3 (0.01)	0 (0.0)
Medications (neuroleptics)	1 (0.0)	0 (0.0)

^a^POI: person of interest.

^b^A single event could contain multiple mentions of a mental illness group (ie, number of mentions).

^c^ICD-10: International Classification of Disease, tenth revision.

### Events With Hospital Records and No Police Mention of Mental Illness

In the 2-year window, 28,777/416,441 (6.91%) domestic violence events had a hospital record but no police mention of mental illness (Table 6), with 15,340/28,777 (53.31%) and 16,793/28,777 (58.36%) events having a hospital record for POIs and victims, respectively. The most prevalent mental illness was mental and behavioral disorders due to psychoactive substance use (9112/15,340, 59.40%, for POIs and 10,017/16,793, 59.65%, for victims), followed by mood disorders (3892/15,340, 25.37%, for POIs and 4070/16,793, 24.24%, for victims; Table 6).

**Table 6:** Number of domestic violence police events with a mention of mental illness for POIs^a^ and victims without a respective hospital record—2-year window period applied^b^.

Mental illness group (ICD-10^c^)	Events (POI; n=15,340)	Events (victim; n=16,793)
Mental and behavioral disorders due to psychoactive substance use	9112 (59.40)	10,017 (59.65)
Mood (affective) disorders	3892 (25.37)	4070 (24.24)
Anxiety, dissociative, stress related, somatoform, and other nonpsychotic mental disorders	3604 (23.49)	3976 (23.68)
Schizophrenia, schizotypal, delusional, and other nonmood psychotic disorders	2627 (17.13)	2789 (16.61)
Disorders of adult personality and behavior	1710 (11.15)	1853 (11.03)
Behavioral and emotional disorders with onset usually occurring in childhood and adolescence	676 (4.41)	708 (4.22)
Unspecified mental disorder	644 (4.20)	641 (3.82)
Intentional self-harm	636 (4.15)	629 (3.75)
Mental disorders due to known physiological conditions	615 (4.01)	724 (4.31)
Symptoms and signs involving cognition, perception, emotional state, and behavior	486 (3.17)	580 (3.45)
Intellectual disability	312 (2.03)	345 (2.05)
Pervasive and specific developmental disorders	239 (1.56)	243 (1.45)
Behavioral syndromes associated with physiological disturbances and physical factors	172 (1.12)	203 (1.21)
Other degenerative diseases of the nervous system	65 (0.42)	58 (0.35)
Systematic atrophies primarily affecting the central nervous system	15 (0.10)	12 (0.07)
Chromosomal abnormalities, not elsewhere classified	12 (0.08)	14 (0.08)
Injury of unspecified body region	1 (0.01)	1 (0.01)

^a^POI: person of interest.

^b^A single event could have several mental illness groups.

^c^ICD-10: International Classification of Disease, tenth revision.

## Discussion

### Principal Findings

These findings build on our previous work, showing that when police attend domestic violence events, they report on a significant number of victims and perpetrators with mental illness [29,31]. Almost 16% (64,587/416,441, 15.51%) of domestic violence events attended by the police had a mention of mental illness [34], which is high considering that this is not proactively probed by the attending officers.

In this study, we explored links between mentions of mental illness in police reports and hospital data. We linked almost half a million police-attended domestic violence events to records from emergency departments (EDDC) and hospital admissions (APDC). Only 3020/416,441 (0.73%) domestic violence events with extracted mental illness information from the police narrative had a corresponding hospital record using a 2-year window period (ie, 12 months before and after the event). This reinforces the view that surveillance systems based solely on hospital presentations are very limited in terms of coverage. We found that concordance (ie, the same mental illness between a police mention and a hospital record for an individual) between these 2 data sources was 15.95% (382/2395) for POI and 16.01% (101/631) for victims. Yet, this lack of concordance between the police and the hospital system is likely explained by the severity of the mental illness, whereby relatively few less severe mental disorders, which may be seen by primary care providers, result in a hospital admission or emergency room attendance.

This does not necessarily reflect poor reporting quality by the NSWPF for several reasons. First, police officers in NSW only undergo a short (1-4 days) period of mental health training as part of their initial training (Chief Inspector Matthew McCarthy, NSWPF, personal communication, July 2021). Second, their primary focus while attending domestic violence events is on victim safety, and thus, recording mental health information is not a priority. Third, recording mental health status is likely to be opportunistic with different motivations in reporting this to the police by victims, perpetrators, and witnesses. The number of police-attended domestic violence events with no mention of mental illness but with a respective hospital record remained roughly the same before (8626 for POIs; 9447 for victims) and after (8157 for POIs; 9804 for victims) the date of the event when a 2-year window period was applied.

Most domestic violence police events with a mental illness mention had no recorded hospital diagnosis within a 2-year window period (59,027/64,587, 91.39%), whereas 83.81% (28,777/34,337) of the hospital admissions that involved a POI or victim had no corresponding mention of mental illness in the police data. Events in which the NSWPF did not record a mental illness mention but had a hospital record could be explained from the 2-year window. It is possible that when police officers attended the event, the individual may not have been diagnosed with a mental illness at that point in time. This can be observed in 28,777/416,441 (6.91%) domestic violence events with a hospital record for POIs and victims, with most of the diagnoses recorded related to substance use (ie, mental and behavioral disorders due to psychoactive substance use), mood, anxiety, and schizophrenic disorders within the 2-year window.

### Concordance With Specific Mental Illnesses

The highest concordance rate between police and health data occurred in events that reported mental and behavioral disorders due to psychoactive substance use for POIs (151 events out of 5616, 2.69%) and victims (26 events out of 1098, 2.37%), with the concordance rate increasing 10-fold when no window period was applied (692/5616, 12.32%, for POIs and 115/1098, 10.47%, for victims). Admissions to residential drug treatment programs within the health system likely explain the higher concordance in terms of diagnoses related to psychoactive substance use. Recent research has demonstrated that substance use (including alcohol consumption) increases both the risk for and impact of domestic violence, highlighting the need for policy that advocates interventions that can address both drug use and violence in combination [45]. These findings have implications about the effect that alcohol and drug abuse might have on the perpetration of domestic violence [46]. Interestingly, unspecified mental disorders were the main recorded group within the police narratives, which is consistent with police’s role not being focused on recording specific details on mental health status.

### Future Implications

Overall, the police appear to have substantial visibility on mental illness in domestic violence, covering the scope and the severity of conditions. Our findings indicated that the NSWPF encounter myriad mental illnesses in the context of domestic violence with more than 120 types identified [32,34]. This further demonstrates the usefulness of employing police narrative data for surveillance and monitoring purposes as we have previously suggested [36].

Partnerships between the police, public health, and welfare sectors with regard to data sharing could help improve domestic violence, health, and justice outcomes, but it often does not occur. For example, data (or algorithm) sharing arrangements could assist in more focused policies and initiatives to improve front-line responses when mental illness is identified, for example, through crisis intervention teams [47-49]. Combining information extracted from domestic violence police records with diagnoses from the health system in privacy preserving ways could help to further explain behaviors and motivations and improve domestic violence prevention. Primary health care providers such as general practitioners (GPs) are also likely to have good visibility on domestic violence for injuries not requiring hospitalization, and less severe forms of mental health and should be considered for linkage as part of a comprehensive data capture system.

No single data collection is likely to have complete coverage of domestic violence, but we have demonstrated that 2 of the big government agencies, the police and secondary and tertiary health care systems (ie, hospitals), can be effectively linked. To improve coverage and identify repeat perpetrators or victims, multiple data sources (eg, GP victim visits vs police attendance) will be required. We identified that only 0.73% (3020/416,441) of domestic violence events had a mental illness recorded in both police records and hospital records, suggesting that other agencies’ data are needed for comprehensive domestic violence reporting.

Interestingly, within the 12-month period, only 841 events for POIs and 919 events for victims (out of 416,441) had a hospital record of mental illness within 48 hours since the domestic violence event occurred. Further, apart from those codes indicating presentations due to psychoactive substance abuse, the recorded codes referred to medical reasons for hospitalization that were unlikely related to domestic violence. This supports the idea that hospitals only see a small fraction of conditions arising from domestic violence situations or do not screen admitted individuals for domestic violence [37,38], which are recognized as limitations of the current domestic violence surveillance [36].

Notwithstanding these factors, we believe that first contact with the police as a consequence of a domestic violence event represents an important opportunity to inquire about the mental health status and enable referral pathways for those requiring treatment and support. This would not be without its challenges but may be important in preventing and minimizing future recurrences of domestic violence. It is possible that these cases of mental illness in domestic violence could be captured from additional data collections that are more appropriate for less severe forms of mental illnesses such as GP visits and psychological services, both of which are covered by Australia’s Medicare Benefits Schedule. However, this requires ongoing dialog and discussion between agencies holding different data collections as well as sophisticated agreements to ensure privacy is protected.

### Limitations

Linking police-recorded domestic violence events with health records from hospital admissions and emergency department presentations has several limitations. There are unique challenges in the appropriate research use of linked administrative data, for example, with respect to bias from linkage errors where records cannot be linked or are linked together incorrectly [28,50]. When linking data sets, a proportion of cases match and a proportion will remain unmatched [50]. The window period of 1 year before and after the date of each event might have limited the number of linked events. Thus, there might have been cases with individuals that were diagnosed after the date when the domestic violence event occurred (eg, after 2 years). Therefore, an expansion of the time frame might return a larger number of linked events (Figure 2) and could potentially assist in providing more population-based information for this type of linkage research and more nuanced conclusions.

Common mental disorders are seen mostly in primary care, while serious mental illness are seen in specialist secondary and tertiary care services. Therefore, common mental disorders are underrepresented in our data. Obtaining and linking other types of clinical data such as GP visits and psychotropic medication prescriptions would embellish the picture of mental health and domestic violence. Police-attended domestic violence events may not result in or be preceded by GP attendances either, and thus remain undetected by the health system. This is a further argument for the benefit of using police data for domestic violence surveillance and employing text mining to extract salient information such as mental illness mentions.

Finally, it is unclear whether any of these extracted mentions of mental illness from police-attended domestic violence events are accurate. These data are not collected for research or reporting purposes and therefore researchers do not get to decide which variables should be recorded [31]. Concordance between police mentions and hospital records with a 2-year window yielded 15.95% (382/2395) for POIs and 16.01% (101/631) for victims, highlighting the need to have a variety of other data sources that can be linked to police records to increase the visibility on the needs of those involved in domestic violence situations, covering law enforcement, social care, health care, welfare.

### Conclusions

Domestic violence is a significant public health problem with its myriad facets being described and recorded in various data collections that cover the health, housing, welfare, justice, child neglect, and finance sectors. However, these different data sources often do not readily share information with each other, preventing the development of a comprehensive picture of domestic violence that can be used for monitoring and prevention purposes. Using a 2-year period to link half a million domestic violence police events with emergency department and hospital admissions, the concordance between the identified mental health mentions from the police narratives and those recorded by the hospital system was 15.95% (382/2395 events) for POIs and 16.0% (101/631 events) for victims. Thus, our findings reinforce the view that surveillance systems based solely on hospital presentations are limited in terms of coverage, with hospitals seeing only a fraction of mental illness conditions arising from domestic violence situations. This demonstrates that police have good visibility of mental illness in domestic violence covering both the scope and the severity of conditions. Despite the set of limitations that the police data come with, the coverage of mental health within the domestic violence police narratives highlights the need to be able to link various data sources for large-scale surveillance and reporting that could prove beneficial for at-risk populations.

## Appendix

Multimedia Appendix 1 Mental illnesses listed in the ICD-10 including the eight new categories targeted for extraction in domestic violence events with examples as they appeared in the police event narratives.

Multimedia Appendix 2 The ICD-10 Mental and Behavioural Disorders schema used to map the extracted mental illness mentions containing three levels (first, second and third).

Multimedia Appendix 3 Fields that describe a police recorded domestic violence event. This information is a combination of the outputs from the application of the text mining methodology on the domestic violence event narratives, the demographic and spatiotemporal information included in the respective fixed fields and the linked mental health diagnoses obtained from the Admitted Patient Data Collection (APDC) and Emergency Department Data Collection (EDDC) datasets. POI refers to the person of interest.

## Abbreviations

APDC: Admitted Patient Data Collection
CHeReL: Centre for Health Record Linkage
CNI: Criminal Number Index
EDDC: Emergency Department Data Collection
ICD-9: International Classification of Disease, ninth revision
ICD-10: International Classification of Disease, tenth revision
NSW: New South Wales
NSWPF: New South Wales Police Force
POI: person of interest
PPN: Project Person Number
SNOMED-CT: Systematized Nomenclature of Medicine - Clinical Terms
