# Supplementary material for: Mental Illness Concordance Between Hospital Clinical Records and Mentions in Domestic Violence Police Narratives: Data Linkage Study
Source: JMIR Form Res. 2022 Oct 20;6(10):e39373. doi: 10.2196/39373 (PMC9634517; doi:10.2196/39373)
Supplement: Multimedia Appendix 3 [file formative_v6i10e39373_app3.docx]

Table 1: Fields that describe a police recorded domestic violence event. This information is a combination of the outputs from the application of the text mining methodology on the domestic violence event narratives, the demographic and spatiotemporal information included in the respective fixed fields and the linked mental health diagnoses obtained from the Admitted Patient Data Collection (APDC) and Emergency Department Data Collection (EDDC) datasets. POI refers to the Person of interest.

| **Name** | **Notes** | **Origin** |
| --- | --- | --- |
| Extracted mental illness - POIs | Extracted mental illness that have been converted into ICD-10 mentions | Text mining domestic violence event narratives |
| Extracted mental illness - victims | Extracted mental illness that have been converted into ICD-10 mentions |  |
| Abuse Type | The type of abuse the POI inflicted on the victim. These include: choking, punching, head locking, assaulting (unspecified), biting, ordering dog attack, dragging, elbowing, grabbing, hair pulling, headbutting, kicking, kneeing, lunging, other (unclassified), physical restraint, sexual assault, pulling, pushing, gagging, scratching, self-harming, shaking, slapping, spitting, stabbing, arm twisting, throwing victim, successful attempt of causing harm with a weapon or object, attempt to use a weapon or object for harm or kill, forced entry into victim’s premises, setting fire on premises or objects, chasing, financial control, stalking, ADVO breach, social restriction, blocking exits or pathways for victim, property damage, preventing child access, harassment, intimidation, miscellaneous, possession of victim’s personal effects, yelling emotional abuse, ADVO breach |  |
| Injury type | Injuries sustained by the victim. These include: bruise, bite marks, black eye, bleeding, burn marks, cut, fracture, graze, lump, miscellaneous, scratch, stab wound, swelling, tearing nails, broken tooth, soreness, redness |  |
| Weapons | Anything used to harm the victim |  |
| Cause | Reason recorded by the NSWPF that started the domestic violence event. It can be one of the following: undefined argument, unknown reasons, use of alcohol and drugs, accusations of infidelity, chores, denied entry to POI, argument involving victim’s or POI’s family, argument involving victim’s or POI’s friends, ignore, late return at home, leave, miscellaneous, request for money, request for POI to move out from premises, loud music, ungiven permission to go out, phone, refusal to do something, relocation of home, termination of relationship, paying rent, request to do something, accusations of seeing ex-partners, accusation of stealing, disposition of POI’s things, victim not wanting POI’s offer in food or things, playing videogames, unemployment |  |
| Threat^a^ | Threat(s) stated by POIs. It can be one of 110 threat statements. Examples are “I want to kill myself,” “I will cut your throat”, “I’ll kill you”, “i will really kill you”, “i will slit you throat, “i will slit you throught”, “i will slit your throat”, “i will stab and shoot you”, “i will stab you and gag you”, “i'll bury you”, “i'll come after your family”, i'll destroy you”, “i'll fucken kill you”, “i'll fucken smash ya” |  |
| Threat Class | Threat classified under certain groups. These include: Direct threat to damage property, direct threat to harm, direct threat to harm third person, direct threat to kill, direct threat to kill third person, direct threat to self-harm, direct threat to sexual assault, direct threat to steal, veiled threat for sexual assault, veiled threat to harm, veiled threat to harm third party, veiled threat to kill, wish for death |  |
| Aboriginal Torres Strait Islander Status (ATSI) - POI | Indigenous status for POI (Yes or No) | NSWPF fixed fields |
| Aboriginal Torres Strait Islander Status (ATSI) - victim | Indigenous status for victim (Yes or No) |  |
| CNI - POI | Criminal Number Index, unique for POI |  |
| CNI - victim | Criminal Number Index, unique for victim |  |
| Year | Year the domestic violence event occurred |  |
| Date of birth - POI |  |  |
| Country of origin - POI |  |  |
| Date of birth - victim |  |  |
| Country of origin - victim |  |  |
| Postcode | Postcode where the domestic violence event occurred |  |
| Suburb | Suburb where the domestic violence event occurred |  |
| Responsible region | The part of the NSWPF that dealt with the DV event. It can include one of the following: Airport Policing, Central Metro, Field Operations, North West Metro, Northern, Operational Information Agency, Police Transport & Public Safety, South West Metro, Southern, Special Services Group, Traffic & Highway Patrol Command, Western |  |
| Responsible local area command | Area covered by the NSWPF stations. |  |
| Sex - POI | Male or female |  |
| Sex - victim | Male or female |  |
| Aboriginal Torres Strait Islander Status (ATSI) ID - POI | Indigenous status for POI (Yes or No) |  |
| Aboriginal Torres Strait Islander Status (ATSI) ID - victim | Indigenous status for victim (Yes or No) |  |
| Premises | Type of premises where the domestic violence event took place. It can be one of the following: adult entertainment, business/commercial, carpark, education, health, industrial, law enforcement, licensed premise, marine transport, outdoor/public place, public transport, recreation, religious, residential, rural industry, transport, unknown, utilities, vehicle) |  |
| Subtype premises* | Subtype of the premises where the domestic violence took place. It can be one of 160 values. Examples are abattoir, gymnasium, hall, homosexual club, rail building, Sydney Entertainment Centre, synagogue, wine bar, |  |
| Further subtype premises* | Further subtype of the premises where the domestic violence took place. It can be one of 300 values. Examples are ambulance station, bus stop, clothing shop, factory, state recreation area, theatre live, workshop |  |
| Homicide | Incident category recorded in a domestic violence event (Yes or No) |  |
| Assault | Incident category recorded in a domestic violence event (Yes or No) |  |
| ADVO breach | Incident category recorded in a domestic violence event (Yes or No) |  |
| Other offence against person | Incident category recorded in a domestic violence event (Yes or No) |  |
| Malicious damage | Incident category recorded in a domestic violence event (Yes or No) |  |
| Common assault | Further classification of the recorded incident category in a domestic violence event (Yes or No) |  |
| Intimidation | Further classification of the recorded incident category in a domestic violence event (Yes or No) |  |
| Actual bodily harm | Further classification of the recorded incident category in a domestic violence event (Yes or No) |  |
| Manslaughter | Further classification of the recorded incident category in a domestic violence event (Yes or No) |  |
| Grievous bodily harm | Further classification of the recorded incident category in a domestic violence event (Yes or No) |  |
| Murder | Further classification of the recorded incident category in a domestic violence event (Yes or No) |  |
| Other offence against other person | Further classification of the recorded incident category in a domestic violence event (Yes or No) |  |
| Kidnapping | Further classification of the recorded incident category in a domestic violence event (Yes or No) |  |
| Malicious damage | Further classification of the recorded incident category in a domestic violence event (Yes or No) |  |
| Domestic | Further classification of the recorded incident category in a domestic violence event (Yes or No) |  |
| Personal | Further classification of the recorded incident category in a domestic violence event (Yes or No) |  |
| Malicious damage with intent | Further classification of the recorded incident category in a domestic violence event (Yes or No) |  |
| Shoot with no intent | Further classification of the recorded incident category in a domestic violence event (Yes or No) |  |
| Shoot with intent pre 4 4 94 | Further classification of the recorded incident category in a domestic violence event (Yes or No) |  |
| Shoot with intent | Further classification of the recorded incident category in a domestic violence event (Yes or No) |  |
| Graffiti | Further classification of the recorded incident category in a domestic violence event (Yes or No) |  |
| Offence against TS | Further classification of the recorded incident category in a domestic violence event (Yes or No) |  |
| Damage public fountain | Further classification of the recorded incident category in a domestic violence event (Yes or No) |  |
| Negligent act | Further classification of the recorded incident category in a domestic violence event (Yes or No) |  |
| Spike food | Further classification of the recorded incident category in a domestic violence event (Yes or No) |  |
| Damage public shrine | Further classification of the recorded incident category in a domestic violence event (Yes or No) |  |
| Assault pre 4 4 94 | Further classification of the recorded incident category in a domestic violence event (Yes or No) |  |
| Bullying/harassment | Further classification of the recorded incident category in a domestic violence event (Yes or No) |  |
| Labour/exploitation | Further classification of the recorded incident category in a domestic violence event (Yes or No) |  |
| Alcohol-related | Associated factor – a tag characterising the domestic violence event in terms of context |  |
| APEC 2007 related | Associated factor – a tag characterising the domestic violence event in terms of context |  |
| Bias Motivation (suspected) | Associated factor – a tag characterising the domestic violence event in terms of context |  |
| Bomb/explosive related | Associated factor – a tag characterising the domestic violence event in terms of context |  |
| Child abuse related | Associated factor – a tag characterising the domestic violence event in terms of context |  |
| Child approach related | Associated factor – a tag characterising the domestic violence event in terms of context |  |
| Domestic violence related | Associated factor – a tag characterising the domestic violence event in terms of context |  |
| Drug abuse related | Associated factor – a tag characterising the domestic violence event in terms of context |  |
| Elder abuse related | Associated factor – a tag characterising the domestic violence event in terms of context |  |
| Firearms related | Associated factor – a tag characterising the domestic violence event in terms of context |  |
| Fraud related | Associated factor – a tag characterising the domestic violence event in terms of context |  |
| Gang related | Associated factor – a tag characterising the domestic violence event in terms of context |  |
| In custody | Associated factor – a tag characterising the domestic violence event in terms of context |  |
| Marine related | Associated factor – a tag characterising the domestic violence event in terms of context |  |
| Mental-illness related | Associated factor – a tag characterising the domestic violence event in terms of context |  |
| Neighbour dispute related | Associated factor – a tag characterising the domestic violence event in terms of context |  |
| No associated factor | Associated factor – a tag characterising the domestic violence event in terms of context |  |
| Non-Domestic Violence related | Associated factor – a tag characterising the domestic violence event in terms of context |  |
| Organised Crime related | Associated factor – a tag characterising the domestic violence event in terms of context |  |
| Other prejudice | Associated factor – a tag characterising the domestic violence event in terms of context |  |
| Personal violence related | Associated factor – a tag characterising the domestic violence event in terms of context |  |
| Police visibility | Associated factor – a tag characterising the domestic violence event in terms of context |  |
| Political prejudice | Associated factor – a tag characterising the domestic violence event in terms of context |  |
| Public place shooting | Associated factor – a tag characterising the domestic violence event in terms of context |  |
| Racial prejudice | Associated factor – a tag characterising the domestic violence event in terms of context |  |
| Religious prejudice | Associated factor – a tag characterising the domestic violence event in terms of context |  |
| Road rage related | Associated factor – a tag characterising the domestic violence event in terms of context |  |
| Rural crime related | Associated factor – a tag characterising the domestic violence event in terms of context |  |
| School related | Associated factor – a tag characterising the domestic violence event in terms of context |  |
| Sexual abuse related | Associated factor – a tag characterising the domestic violence event in terms of context |  |
| Sexual preference prejudice | Associated factor – a tag characterising the domestic violence event in terms of context |  |
| Terrorism related | Associated factor – a tag characterising the domestic violence event in terms of context |  |
| Transit related | Associated factor – a tag characterising the domestic violence event in terms of context |  |
| World Youth Day (WYD) 2008 | Associated factor – a tag characterising the domestic violence event in terms of context |  |
| Date of the event |  |  |
| Agricultural grain | Weapon Category (Yes or No) |  |
| Air pistol | Weapon Category (Yes or No) |  |
| Air rifle | Weapon Category (Yes or No) |  |
| Vending machine | Weapon Category (Yes or No) |  |
| Animal | Weapon Category (Yes or No) |  |
| Animal related | Weapon Category (Yes or No) |  |
| Electrical appliance | Weapon Category (Yes or No) |  |
| Other appliance | Weapon Category (Yes or No) |  |
| Art/craft instrument | Weapon Category (Yes or No) |  |
| Sculpture | Weapon Category (Yes or No) |  |
| Award | Weapon Category (Yes or No) |  |
| Bike | Weapon Category (Yes or No) |  |
| Book | Weapon Category (Yes or No) |  |
| Boomerang | Weapon Category (Yes or No) |  |
| Bow and arrow | Weapon Category (Yes or No) |  |
| Rock | Weapon Category (Yes or No) |  |
| Clock | Weapon Category (Yes or No) |  |
| Clothes | Weapon Category (Yes or No) |  |
| Iron bar | Weapon Category (Yes or No) |  |
| Coin, card, stamp | Weapon Category (Yes or No) |  |
| Communication equipment | Weapon Category (Yes or No) |  |
| Computer hardware | Weapon Category (Yes or No) |  |
| Drug | Weapon Category (Yes or No) |  |
| Drug implement | Weapon Category (Yes or No) |  |
| Explosive | Weapon Category (Yes or No) |  |
| Firearm equipment | Weapon Category (Yes or No) |  |
| Firearm | Weapon Category (Yes or No) |  |
| Firearm, no description | Weapon Category (Yes or No) |  |
| Firearm, unseen | Weapon Category (Yes or No) |  |
| Weapon, no description | Weapon Category (Yes or No) |  |
| Weapon, unseen | Weapon Category (Yes or No) |  |
| Fist | Weapon Category (Yes or No) |  |
| Ramset | Weapon Category (Yes or No) |  |
| Fuel | Weapon Category (Yes or No) |  |
| Furniture | Weapon Category (Yes or No) |  |
| Garden equipment | Weapon Category (Yes or No) |  |
| Glass | Weapon Category (Yes or No) |  |
| Hammer | Weapon Category (Yes or No) |  |
| Handgun, no description | Weapon Category (Yes or No) |  |
| Hardware | Weapon Category (Yes or No) |  |
| Entertainment system equipment | Weapon Category (Yes or No) |  |
| Home item | Weapon Category (Yes or No) |  |
| Jewellery | Weapon Category (Yes or No) |  |
| Chain | Weapon Category (Yes or No) |  |
| Ring | Weapon Category (Yes or No) |  |
| Knife | Weapon Category (Yes or No) |  |
| Leisure equipment | Weapon Category (Yes or No) |  |
| Beer, wine | Weapon Category (Yes or No) |  |
| Spirits | Weapon Category (Yes or No) |  |
| Luggage | Weapon Category (Yes or No) |  |
| Machine Gun | Weapon Category (Yes or No) |  |
| Measuring instrument | Weapon Category (Yes or No) |  |
| Medical item | Weapon Category (Yes or No) |  |
| Metal | Weapon Category (Yes or No) |  |
| Miscellaneous | Weapon Category (Yes or No) |  |
| Music Acc | Weapon Category (Yes or No) |  |
| Sting instrument | Weapon Category (Yes or No) |  |
| Woodwind instrument | Weapon Category (Yes or No) |  |
| Office equipment | Weapon Category (Yes or No) |  |
| Capsicum spray | Weapon Category (Yes or No) |  |
| Other | Weapon Category (Yes or No) |  |
| Firearm, other | Weapon Category (Yes or No) |  |
| Military firearm | Weapon Category (Yes or No) |  |
| Prohibited firearm | Weapon Category (Yes or No) |  |
| Paint | Weapon Category (Yes or No) |  |
| Personal item | Weapon Category (Yes or No) |  |
| Pesticide | Weapon Category (Yes or No) |  |
| Photo equipment | Weapon Category (Yes or No) |  |
| Pistol | Weapon Category (Yes or No) |  |
| Prohibited article | Weapon Category (Yes or No) |  |
| Pump | Weapon Category (Yes or No) |  |
| Rep firearm | Weapon Category (Yes or No) |  |
| Revolver | Weapon Category (Yes or No) |  |
| Rifle | Weapon Category (Yes or No) |  |
| Rope | Weapon Category (Yes or No) |  |
| Sawn-off gun | Weapon Category (Yes or No) |  |
| Sharp | Weapon Category (Yes or No) |  |
| Short rifle | Weapon Category (Yes or No) |  |
| Shotgun | Weapon Category (Yes or No) |  |
| SB shotgun | Weapon Category (Yes or No) |  |
| Speargun | Weapon Category (Yes or No) |  |
| Ball | Weapon Category (Yes or No) |  |
| Gym equipment | Weapon Category (Yes or No) |  |
| Indoor equipment | Weapon Category (Yes or No) |  |
| Outdoor equipment | Weapon Category (Yes or No) |  |
| Water equipment | Weapon Category (Yes or No) |  |
| Syringe | Weapon Category (Yes or No) |  |
| Textile | Weapon Category (Yes or No) |  |
| Tobacco | Weapon Category (Yes or No) |  |
| Tool | Weapon Category (Yes or No) |  |
| Toy | Weapon Category (Yes or No) |  |
| Pram | Weapon Category (Yes or No) |  |
| Vehicle Acc | Weapon Category (Yes or No) |  |
| Vehicle panel | Weapon Category (Yes or No) |  |
| Vehicle mechanics | Weapon Category (Yes or No) |  |
| Number-plate | Weapon Category (Yes or No) |  |
| Unrecorded weapon | Weapon not stated but not found |  |
| Glass bottle | Weapon Category (Yes or No) |  |
| Probable non-weapon | Weapon Category (Yes or No) |  |
| NULL | No weapon indicated, recorded or found |  |
| Sharp instrument | Weapon Category (Yes or No) |  |
| Blunt instrument | Weapon Category (Yes or No) |  |
| Other weapon | Weapon Category (Yes or No) |  |
| Long arm | Weapon Category (Yes or No) |  |
| Short arm | Weapon Category (Yes or No) |  |
| Replica | Weapon Category (Yes or No) |  |
| Other firearm | Weapon Category (Yes or No) |  |
| Carer | POI relationship to victim |  |
| Sibling | POI relationship to victim |  |
| Perpetrator unknown to victim | POI relationship to victim |  |
| Parent guardian of victim | POI relationship to victim |  |
| Person in authority | POI relationship to victim |  |
| Relationship unknown or unstated | POI relationship to victim |  |
| Child of victim, including step or foster | POI relationship to victim |  |
| Other, known person of no relationship | POI relationship to victim |  |
| Other family member | POI relationship to victim |  |
| Boyfriend/girlfriend, including ex | POI relationship to victim |  |
| Household member, including former | POI relationship to victim |  |
| Ex-spouse/partner | POI relationship to victim |  |
| Spouse/partner | POI relationship to victim |  |
| Not applicable | POI relationship to victim |  |
| Not recorded | POI relationship to victim |  |
| ICD-10 mental illness code | Mental illness diagnosis code from APDC, EDDC or both | NSW Health |
| POI/Victim | Indicator for whether the code in the previous field is for victim or POI |  |

^a^in a field indicates that only some examples are provided due to the large number of potential values that they can have. The ICD-10 mental illness code (NSW Health) field can have more than one code describing a mental illness for a POI or a victim.
